# Decreased resting-state functional connectivity of the habenula-cerebellar in a major depressive disorder

**DOI:** 10.3389/fpsyt.2022.925823

**Published:** 2022-09-06

**Authors:** Ju-Yeon Jung, Seo-Eun Cho, Nambeom Kim, Chang-Ki Kang, Seung-Gul Kang

**Affiliations:** ^1^Department of Health Science, Gachon University Graduate School, Incheon, South Korea; ^2^Department of Psychiatry, Gil Medical Center, Gachon University College of Medicine, Incheon, South Korea; ^3^Department of Biomedical Engineering Research Center, Gachon University, Incheon, South Korea; ^4^Department of Radiological Science, College of Health Science, Gachon University, Incheon, South Korea

**Keywords:** major depressive disorder, restring state functional connectivity, habenula, septal nuclei, cerebellum, subcortical areas

## Abstract

**Background:**

In animal experiments, the habenula and septal nuclei are known as the key brain areas of depression. However, there are few magnetic resonance imaging (MRI) studies on the functional connectivity between these areas and the subcortical areas in humans with major depression. We aimed to investigate the difference in resting-state functional connectivity (RSFC) among the major regions of interest (ROI) in the subcortical areas, including both the habenula and septal nuclei.

**Methods:**

We performed the seed-to-voxel analysis to investigate the RSFC between both the habenula and septal nucleus, as well as other subcortical regions. Furthermore, ROI-to-ROI analysis was performed among the combinations of ROI pairs in the subcortical areas.

**Results:**

The seed-to-voxel analysis showed a lower RSFC between the left habenula and the cerebellum in major depressive disorder (MDD) than in healthy controls (HCs). As a result of ROI-to-ROI analysis in subcortical areas, a total of 31 pairs of FCs in the MDD group showed a lower RSFC than in the HCs group.

**Conclusion:**

This study revealed a lower RSFC between the left habenula and cerebellum in patients with MDD and reduced RSFC among numerous subcortical areas. These new findings on the neural circuitry of MDD might contribute to an in-depth understanding of depression.

## Introduction

Major depressive disorder (MDD) is one of the most common and disabling mental illnesses and is known to impair patients’ quality of life (1). Many factors related to the occurrence of depression are genetic factors, stressors, traumatic events, parenting, abuse, loss, gender, and physical illness; however, recently, depression has been considered a brain disease (2). For this reason, many studies on brain structure have been performed to find the etiology of MDD, and the abnormalities of many brain areas, including the anterior cingulate, prefrontal and orbitofrontal areas, amygdala, and hippocampus, have been reported (3, 4). In addition, numerous functional neuroimaging studies have found abnormalities in the brain dysfunction of MDD (4–7).

The habenula is a small (5–9 mm in diameter) and bilateral epithalamic structure located in the midbrain and is known to be important for brain signaling and learning from negative events, the reward system, motivational and emotional control of behavior, and stress response (8–11). This area has been reported to be related to depression in many animal experiments. It has been reported to have a strong relationship with psychiatric conditions such as depression and suicidality in human studies (12–15). In addition, several brain imaging studies have also reported abnormal findings of the habenula in depression (16–18). The septal nuclei are adjacent brain regions to the hippocampus, dorsal raphe nucleus, hypothalamus, and habenula and are known to be involved in emotion, memory, and learning, as well as feeding behavior (19). Although the septal nucleus is known as one of the key areas of depression in animal experiments (20), very few *in vivo* brain imaging studies have been conducted in humans in this region. The postmortem morphometric analysis, which was rarely performed on humans, showed a significant negative correlation between the neuronal density of the lateral septal nucleus and disease duration in MDD (21). Both habenula and septal nuclei have been reported to regulate mood-related neurotransmitters by linking with monoamine centers in animal experiments (22); however, there are few studies on this in humans.

Recently, as the *in vivo* fMRI investigation into the human habenula began, Ely et al. succeeded in mapping the whole-brain resting-state functional connectivity (RSFC) of the human habenula in healthy young adults (23). In particular, they identified significant positive habenula connectivity with brainstem targets and subcortical structures, including the ventral tegmental area, dorsal raphe, thalamus, and cerebellum (23). The patients diagnosed with a mood disorder (MDD or bipolar disorder) displayed higher RSFC between the left habenula and angular gyrus, middle temporal gyrus, and posterior cingulate, as well as decreased RSFC between the right habenula and left thalamus (15).

In the past, there have been many studies on cortical areas such as the prefrontal area and the anterior cingulate cortex for the pathogenesis of depression (24); however, recently, there have been increasing reports of the importance of the brainstem and subcortical areas such as the thalamus and cerebellum in the pathogenesis of depression (25). There have been many studies suggesting that the structure, function, and connectivity of the brainstem, thalamus, and cerebellum are abnormal in depression (5, 26–35). The areas such as the locus ceruleus, dorsal raphe, and ventral tegmental area that secrete neurotransmitters related to depression (norepinephrine, serotonin, and dopamine) exist in the brainstem (36, 37), and the thalamus is considered to be one of the major brain regions involved in the pathophysiology of depression, emotions, and restorative autonomic and endocrine processes (38). The cerebellum is known to be involved in the various aspects of cognition and affect beyond the motor domain and in the MDD pathophysiology communicating with the cortical networks sub serving the self-referential and cognitive processing (39). However, few studies have been conducted on the functional connections between these domains or between these domains and the habenula and septal nuclei.

Resting-state fMRI (rs-fMRI) is an outstanding method to probe neural networks, as it shows the RSFC between the brain regions by measuring the blood oxygen level-dependent signal during rest (40). Positive and negative correlations between two areas are considered to reflect synchrony in regions functioning toward similar and opposite goals, respectively (41). We hypothesized that the functional connectivity between the habenula, cerebellum, septal nuclei, and other subcortical structures would be lower in the MDD group than in the control group, and we sought to find networks with particularly different connectivity between each structure. Therefore, we attempted to study RSFC among the major regions of interest (ROI) in the subcortical areas (i.e., brainstem, thalamus, and cerebellum), including both the habenula and septal nuclei. The aims of this study were (1) to investigate whether the RSFC between pre-defined seeds (habenula and septal nuclei) and other subcortical areas would differ between MDD and control groups (seed-to-voxel analysis) and (2) to investigate whether the RSFC among the combinations of ROI pairs in the subcortical areas differs between the two groups (ROI-to-ROI analysis).

## Materials and methods

### Participants and clinical measurement

Forty-six patients with MDD and 38 healthy controls (HC) participated in this study. All participants provided written informed consent to participate in the study. This study was approved by the Institutional Review Board of the Gil Medical Center (IRB No. GDIRB2018-005 and GDIRB2020-207). One board-certified psychiatrist (SGK) interviewed all the participants and assessed their eligibility for this study using a Structured Clinical Interview for the fifth edition of the Diagnostic and Statistical Manual of Mental Disorders (DSM-5) (SCID-5) (42). Patients meeting the DSM-5 diagnostic criteria for MDD were included in the MDD group (43).

The following common exclusion criteria were applied: age under 20 or over 65 years, left-handed using the Edinburgh Handedness Test (44), unstable or major medical condition, neurological disorders within the past 1 year, substance use disorder within the past 1 year, intellectual disability, personality disorder, current serious suicidal risk, neurocognitive disorders, history of head trauma, previous abnormal findings in brain imaging, contraindications to magnetic resonance imaging (MRI) (e.g., metals in the body), and pregnancy or lactation. Additional exclusion criteria for MDDs were the comorbidities of major psychiatric disorders: schizophrenia spectrum and other psychotic disorders, major anxiety disorders, obsessive-compulsive and related disorders, substance-related and addictive disorders, and disruptive, impulse-control, and conduct disorders. Additional exclusion criteria were added for HCs: any psychiatric history, Hamilton Depression Rating Scale 17 items (HDRS-17) total score > 6, history of taking psychotropic medications, and first-degree relatives with major psychiatric disorders, such as schizophrenia, MDD, or bipolar disorders.

Depression severity was quantified using the HDRS-17 (45), Clinical Global Impression of Severity (CGI-S) (46), and Beck Depression Inventory (BDI) (47, 48) at baseline and MRI scanning date. Based on the HDRS-17 score, the severity of depression was classified as follows: severe depression (≥25), moderate depression (18–24), mild depression (7–17), and no depression (0–6) (49). We assessed depressive symptoms on the same day we scanned the brain image.

### Data acquisition

Functional and anatomical images were acquired using a 3T MRI system (Siemens Verio or Vida, Erlangen, Germany) with a 20-channel radiofrequency head coil. For functional images, participants were asked to close their eyes, stay awake, and not perform any head motions until the scan was performed, and the inside of the scanner was monitored in real time. Participants also verbally answered questions about their condition between the scans. For data acquisition, two-dimensional echo planar imaging (EPI) was used with the following parameters: repetition time (TR)/echo time (TE)/acquisition time (TA) = 2,500 ms/25 ms/6 min 45 s, field of view (FOV) = 231 mm, flip angle (FA) = 90°, in-plane resolution = 3.5 × 3.5 mm 2, slice thickness = 3.5 mm, slices = 42, and measurements = 160. For anatomical image acquisition, T1-weighted anatomical three-dimensional imaging with magnetization-prepared rapid acquisition gradient echo (MPRAGE) was used with the following parameters: TR/TE/inversion time (TI)/TA = 1,900 ms/3.3 ms/900 ms/3 min 40 s, FOV = 256 mm, FA = 9°, in-plane resolution = 1.0 × 1.0 mm2, slice thickness = 1 mm, slices = 160.

### Data processing

All data were preprocessed by using the MATLAB-based CONN functional connectivity toolbox ver. 18b.^1^ CONN is used for the correlation analysis of RSFC in fMRI. Prior to preprocessing, we discarded the first five volumes (12.5 s) and the last five volumes (12.5 s) out of 160 volumes in each group. The first five volumes were discarded to ensure only the collection of stabilized data, and the last five volumes were discarded because the data from one of the participants was accidentally omitted from the last five volumes. In the preprocessing analyses, 150 functional volumes were utilized for preprocessing analysis. Preprocessing included realignment to the first volume for slice timing correction, co-registration with anatomical images, segmentation, and normalization to the Montreal Neurological Institute (MNI) with a resampling voxel size of 2 × 2 × 2 m^3^. Finally, functional images were smoothed by a Gaussian kernel with a full width at half maximum (FWHM) of 8 mm.

The imported 71 ROIs for FC analysis were 68 subcortical ROIs, bilateral habenula and septum. The 68 ROIs include the thalamus (30 ROIs), cerebellum (18 ROIs), vermis (8 ROIs), and brainstem (12 ROIs) of automated anatomical labeling (AAL) version 3 (50). The AAL is the most widely used brain parcellation map that includes both structural and functional areas. Notably, the latest version (AAL3) includes more subtle ROIs in the brainstem, such as the red nucleus (50). Bilateral habenula and septal areas not included in the AAL3 were manually segmented on single-subject high-resolution T1 volume images (Supplementary Figure 1) (51, 52). An experienced researcher first performed the ROI segmentation using the MRIcron software^2^; thereon, another senior researcher double-checked the size and location of the segmented ROIs.

### Functional connectivity analysis and statistical analysis

The preprocessed fMRI data were used to perform the RSFC analyses. All data were band-pass filtered (0.008–0.09 Hz), and artifact Detection Tool (ART)-based scrubbing was performed to detect outliers. Head motion outliers were defined as movements of distances greater than 2.5 mm or angles greater than 2.5° (53). The group difference in head motion was assessed using the average frame wise displacement (FD) between groups to estimate subject-specific movements over time (54). There was no significant difference in FD between groups (*t* = –1.397; *p* = 0.166). Denoising procedures to remove physiological and other spurious noise sources were implemented in the anatomical component-based noise correction (CompCor) strategy. A seed-to-voxel analysis was performed to identify functional correlations between the segmented regions (both habenula and septal nucleus) and subcortical regions. The connectivity between the seeds (left and right habenular and septal nuclei) and subcortex regions was measured for the seed-to-voxel analysis. The subcortical regions of 71 ROIs included 68 ROIs extracted from the AAL3, bilateral habenula, and septal nucleus. Furthermore, ROI-to-ROI analysis was also performed to identify functional correlations between subcortical regions. To apply a group-level analysis, Pearson’s correlation coefficients were converted to *z*-scores using Fisher’s r-to-z transformation. An independent *t*-test was used to compare differences between the normal group and MDD. Multiple comparisons were corrected by a false discovery rate (FDR) (55). Seed-to-voxel results were thresholded at an FDR-corrected cluster level of *p* < 0.05, while an uncorrected peak level of *p* < 0.001 was used for each seed. An ROI-to-ROI analysis with 71 ROIs was performed using two-sample *t*-test statistics. ROI-to-ROI results were thresholded at an FDR seed level corrected to *p* < 0.05.

Demographic and clinical data were analyzed and compared between two groups using a Student’s *t*-test or chi-square test. The statistical analyses for the clinical data were performed at a two-sided significance level of *p* < 0.05 using the Statistical Package for the Social Sciences (SPSS, IBM Inc.) program.

## Results

A total of 84 participants (46 patients with MDD and 38 HCs) were included in the analysis. Most patients with MDD in this study were outpatients and inpatients of the Department of Psychiatry and the Gil Medical Center. The participants in the control group and some patients with MDD were contacted after they responded to the study recruitment notice posted in the hospital. Their demographics, psychiatric history, and clinical characteristics are presented in Table 1. There was no significant difference in age or proportion of females between the two groups. The HDRS-17 scores were significantly higher (*p* < 0.001) in the MDD group than in the HCs group. Furthermore, patients with MDD had significantly higher BDI, BAI, BHS, and CGI-S scores than normal controls (*p* < 0.001, see Table 1). When the severity of the MDD group was classified according to the HDRS-17, the number of participants classified as severe, moderate, mild, and normal were 8 (17%), 15 (33%), 20 (43%), and 3 (7%), respectively. Additionally, in the MDD group, the number of cases classified as recurrence, the first episode, treatment resistance, and remission were 18 (39%), 22 (48%), 9 (20%), and 2 (4%), respectively. Some cases belonged to two or more subgroups; therefore, the total percentage exceeded 100%. In the MDD group, the mean duration of illness was 5.8 years, and the average duration of the current episode was 69.4 weeks (Table 1).

**TABLE 1:** Demographic and clinical characteristics and their comparison between MDD and HC groups.

Variables	MDD (*n* = 46)	HC (*n* = 38)	Statistical tests	
			
			*t* or *x*^2^	*P*
**Demographics**				
Age, years^a^	38.3 ± 12.5	37.1 ± 13.0	*t* = –0.45	0.655^a^
Sex, female, *N* (%)^b^	35 (76.1)	26 (68.4)	*x*^2^ = 0.62	0.433^b^
**Psychiatric history**				
Duration of illness, years	5.8 ± 5.9	N/A	N/A	N/A
Duration of current episode, weeks	69.4 ± 77.9	N/A	N/A	N/A
The use of antidepressants, *N* (%)	40 (87.0)	N/A	N/A	N/A
Duration of taking antidepressants, years	3.7 ± 3.6	N/A	N/A	N/A
**Severity ranges for the HDRS-17 score**				
Severe depression (≥25), *N* (%)	8 (17.4)	0	N/A	N/A
Moderate depression (18–24), *N* (%)	15 (32.6)	0	N/A	N/A
Mild depression (7–17), *N* (%)	20 (43.5)	0	N/A	N/A
Normal range (0–6), *N* (%)	3 (6.5)	38 (100)	N/A	N/A
**MDD subgroups** *				
Recurrence, *N* (%)	18 (39.1)	N/A	N/A	N/A
Treatment-resistance, *N* (%)	9 (19.6)	N/A	N/A	N/A
The first episode, *N* (%)	22 (47.8)	N/A	N/A	N/A
Remission, *N* (%)	2 (4.3)	N/A	N/A	N/A
**Main types of antidepressants**				
Selective serotonin reuptake inhibitors, *N* (%)	23 (50.0)	N/A	N/A	N/A
Serotonin-norepinephrine reuptake inhibitors, *N* (%)	6 (13.0)	N/A	N/A	N/A
Vortioxetine, *N* (%)	3 (6.5)	N/A	N/A	N/A
Mirtazapine, *N* (%)	3 (6.5)	N/A	N/A	N/A
Agomelatine, *N* (%)	3 (6.5)	N/A	N/A	N/A
Bupropion, *N* (%)	2 (4.3)	N/A	N/A	N/A
**Clinical scales** ^a^				
HDRS-17	16.5 ± 6.1	2.6 ± 2.4	*t* = –14.16	<0.001^a^
BDI	28.2 ± 13.1	3.5 ± 3.6	*t* = –12.26	<0.001^a^
BAI	25.2 ± 15.7	2.8 ± 4.6	*t* = –9.22	<0.001^a^
BHS	11.6 ± 5.2	2.3 ± 1.8	*t* = –11.39	<0.001^a^
CGI-S	4.1 ± 1.1	1.1 ± 0.2	*t* = –18.38	<0.001^a^

Data are presented as means ± standard deviations or numbers (percentages). Statistical tests were performed using ^a^Student’s t-test or ^b^Chi-square test.

*Some cases belonged to two or more subgroups.

BAI, Beck Anxiety Inventory; BDI, Beck Depression Inventory; BHS, Beck Hopelessness Scale; CGI-S, Clinical Global Impression-Severity; HC, healthy control; HDRS-17, 17-item version of the Hamilton Depression Rating Scale; MDD, Major Depressive Disorder.

87.0% of the participants with MDD were taking antidepressants, and the average duration of taking antidepressants was 3.7 years. The main types of antidepressants used in the MDD group were selective serotonin reuptake inhibitors (*n* = 23), serotonin-norepinephrine reuptake inhibitors (*n* = 6), vortioxetine (*n* = 3), mirtazapine (*n* = 3), agomelatine (*n* = 3), and bupropion (*n* = 2). Twenty-seven patients took only one antidepressant, 11 patients took two antidepressants, and two patients took three antidepressants. In addition to antidepressants, other psychotropic medicines used were benzodiazepines (*n* = 21) and aripiprazole (*n* = 6).

**FIGURE 1:**
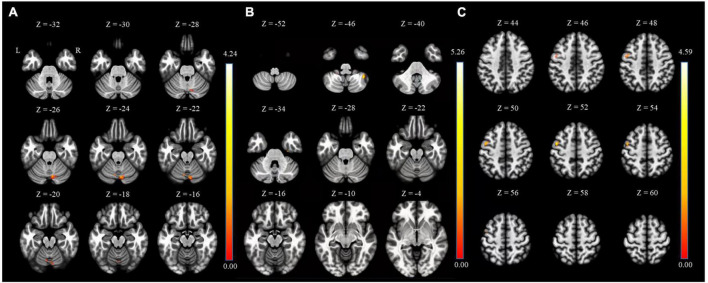
Regions of HC > MDD functional connectivity in the seed-to-voxel analysis. **(A)** L-Cerebellum_6 connected with L-Habe seed (MNI coordinates: X = –08, Y = –66, Z = –22), **(B)** R-Cerebellum_7b connected with R-Habe seed (MNI coordinates: X = + 40, Y = –50, Z = –46) but not significant on statistically. **(C)** PreCG_L connected with septal seed (MNI coordinates: X = –46, Y = + 00, and Z = + 52) but not significant on statistically. HC, Healthy controls; L-Cerebellum_6, Lobule VI of the left cerebellar hemisphere; L-Habe, Left habenula; MDD, Major depressive disorder; MNI, Montreal Neurological Institute; R-Cerebellum_7b, Lobule VIIB of the right cerebellum; R-Habe, Right habenula.

Between HC and the MDD groups, FC analysis was performed in two types: seed-to-voxel and ROI-to-ROI analysis. As shown in Figure 1 and Table 2, the results of the seed-to-voxel analysis with both habenula and septal nucleus as seeds showed a significantly lower RSFC between the left habenula (L-Habe) seed and the area that includes lobule VI of the left cerebellar hemisphere (L-Cerebellum_6, 63 voxels), lobule VII of the vermis (Vermis_7, 56 voxels), and lobule VI of the right cerebellar hemisphere (R-Cerebellum_6, 34 voxels) (FDR-corrected *p* = 0.001) in MDD than HCs. However, in the analysis using the right habenula (R-Habe) as a seed, there was an evident difference in the RSFC of lobule VIIB of the right cerebellum (R-Cerebellum_7b) (uncorrected *p* = 0.039) between the groups (HC > MDD). No region showed significant FC between the groups with the septal nucleus (Sep_N) as a seed. Additionally, in the seed-to-voxel analysis, there was no area in which the MDD group showed significantly higher FC than the HC group.

**TABLE 2:** Seed-to-voxel connectivity results with both habenula seeds.

Contrast	Seed	Cluster			Peak			Peak MNI coordinates			AAL label
					
		p (FDR)	p (unc.)	K	T	Z	p (unc.)	X	Y	Z	
HC > MDD	L-Habe	0.001*	0.000	255	4.32	4.09	0.000	–08	–66	–22	L-Cerebellum_6
					4.29	4.06	0.000	+ 4	–72	–24	Vermis_7
					3.46	3.33	0.000	+ 14	–68	–24	R-Cerebellum_6
	R-Habe	0.394	0.039	58	4.58	4.31	0.000	+ 40	–50	–46	R-Cerebellum_7b

The largest cluster (K) regions in each seed were indicated as a representative. Bold value indicate statistical significance (*p < 0.05).

AAL, Automated Anatomical Labeling; FDR-corr, false discovery rate corrected; HC, Healthy controls; L-Cerebellum_6, Lobule VI of the left cerebellar hemisphere; L-Habe, Left habenula; MDD, Major Depressive Disorder; MNI, Montreal Neurological Institute; R-Cerebellum_6, Lobule VI of right cerebellum; R-Cerebellum_7b, Lobule VIIB of the right cerebellum; R-Habe, right habenula; unc., uncorrected.

**FIGURE 2:**
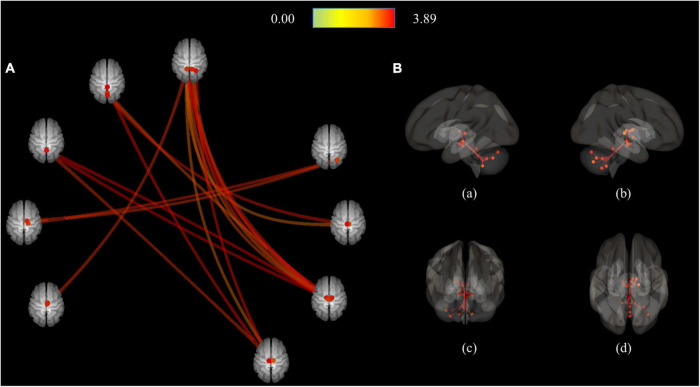
Connection pairs of HC > MDD functional connectivity within 71 ROIs for the ROI-to-ROI analyses. **(A)** ROI-to-ROI connectome ring maps **(B)** ROI-to-ROI 3D rendering maps on the left (a), right (b), anterior (c), and superior view (d). The color bar indicates the statistical *T*-value. HC, Healthy controls; MDD, Major depressive disorder; ROI, Region of interest.

**FIGURE 3:**
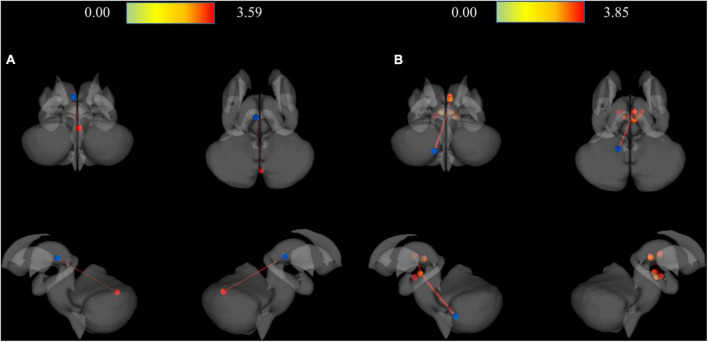
3D rendering maps of ROI-to-ROI connection pairs in the subcortical regions. **(A)** Functional connection of L-Habe with Vermis_7 target presented on posterior, superior, left, and right views. Blue and red spots represent the L-Habe seed and the Vermis_7 target. **(B)** Functional connection of L-Cerevellum_9 seed with R-SN_pr, L-SN-pr, R-Thal_MDm, R-Red_N, L-Red_N, R-SN-pc, and R-Habe targets presented on posterior, superior, left and right views. Blue and red spots represent the L-Cerebellum_9 seed and seven targets, respectively. HC, Healthy controls; L-Cerebellum_9, Lobule IX of the left cerebellum; L-Habe, Left habenula; L-Red_N, Left red nucleus; L-SN_pr, Left substantia nigra, pars reticulata; MDD, Major depressive disorder; R-Habe, Right habenula; ROI, Region of interest; R-Red_N, Right red nucleus; R-SN_pc, Right substantia nigra, pars compacta; R-SN_pr, Right substantia nigra, pars reticulate; R-Thal_MDm, Right mediodorsal medial magnocellular thalamus; Vermis_7, Lobule VII of the vermis.

As a result of ROI-to-ROI analysis in subcortical areas, a total of 31 pairs of FCs in the MDD group showed a lower RSFC than the HCs group. Vermis_9–right red nucleus (R-Red_N) showed the most significant difference in RSFC (FDR-corrected *p* = 0.0048) between the groups. The lobule IX of the left cerebellum (L-Cerebellum_9) showed connectivity with the most target ROIs, including the following areas: right substantia nigra pars reticulate (R-SN_pr), left substantia nigra pars reticulate (L-SN_pr), right mediodorsal medial magnocellular thalamus (R-Thal_MDm), right red nucleus (R-Red_N), left red nucleus (L-Red_N), right substantia nigra, pars compacta (R-SN_pc) and right habenula (R-Habe), as shown in Figure 2 and Table 3. Furthermore, both habenula areas showed significant connectivity with Vermis_7 (L-Habe-Vermis_7) and L-Cerebellum_9 (L-Cerebellum_9–R-Habe) (Figure 3).

**TABLE 3:** Connected pairs showing significant differences in functional connectivity on HC > MDD contrast.

Connection pairs			Statistic T (df = 82)	p-FDR
		
Seed regions		Target regions		
R-Thal_VPL	–	R-Cerebellum_Crus2	3.44	0.0322
R-Thal_MDm	–	L-Cerebellum_9	3.28	0.0388
R-Thal_MDm	–	R-Thal_Re	3.16	0.0388
L-VTA	–	R-Cerebellum_8	3.62	0.0177
L-Habe	–	Vermis_7	3.59	0.0198
L-SN_pc	–	Vermis_1_2	3.65	0.0163
L-SN_pr	–	L-Cerebellum_9	3.54	0.0231
R-SN_pr	–	L-Cerebellum_9	3.85	0.008
L-Red_N	–	Vermis_9	3.8	0.0097
L-Red_N	–	R-Cerebellum_9	2.96	0.0495
L-Red_N	–	Vermis_8	2.94	0.0495
L-Red_N	–	L-Cerebellum_9	2.84	0.0495
R-Red_N	–	Vermis_9	3.89	0.0071
R-Red_N	–	R-Cerebellum_9	3.35	0.0216
R-Red_N	–	L-Cerebellum_9	3.02	0.0399
Vermis_1_2	–	L-SN_pc	3.65	0.0163
Vermis_7	–	L-Habe	3.59	0.0198
Vermis_9	–	R-Red_N	3.89	0.0048
Vermis_9	–	L-Red_N	3.8	0.0048
Vermis_9	–	R-SN_pc	3.29	0.0173
R-Cerebellum_Crus2	–	R-Thal_VPL	3.44	0.0315
R-Cerebellum_Crus2	–	R-Thal_VL	3.23	0.0315
R-Cerebellum_8	–	L-VTA	3.62	0.0177
L-Cerebellum_9	–	R-SN_pr	3.85	0.008
L-Cerebellum_9	–	L-SN_pr	3.54	0.0116
L-Cerebellum_9	–	R-Thal_MDm	3.28	0.0177
L-Cerebellum_9	–	R-Red_N	3.02	0.0299
L-Cerebellum_9	–	L-Red_N	2.84	0.0396
L-Cerebellum_9	–	R-SN_pc	2.64	0.0491
L-Cerebellum_9	–	R-Habe	2.64	0.0491
R-Cerebellum_9	–	R-Red_N	3.35	0.0431

df; degree of freedom; FDR-corr, false discovery rate corrected; HC, healthy controls; L-Habe, left habenula; L-Cerebellum_9, Lobule IX of the left cerebellum; L-Red_N, left red nucleus; L-SN_pc, left substantia nigra, pars compacta; L-SN_pr, Left substantia nigra, pars reticulata; L-VTA, left ventral tegmental area; MDD, major depressive disorder; R-Cerebellum_Crus2, Crus II of the right cerebellum; R-Cerebellum_8, Lobule VIII of right cerebellum; R-Cerebellum_9, Lobule IX of the right cerebellum; R-Habe, right habenula; R-Red_N, right red nucleus; R-SN_pc, the right substantia nigra, pars compacta; R-SN_pr, Right substantia nigra, pars reticulata; R-Thal_MDm, right mediodorsal medial magnocellular thalamus; R-Thal_Re, Right reuniens thalamus; R-Thal_VL, right ventral lateral thalamus; R-Thal_VPL, right ventral posterolateral thalamus; Vermis_1_2, Lobule I, II of vermis; Vermis_7, Lobule VII of vermis; Vermis_8, Lobule VIII of vermis; Vermis_9, Lobule IX of vermis.

**FIGURE 4:**
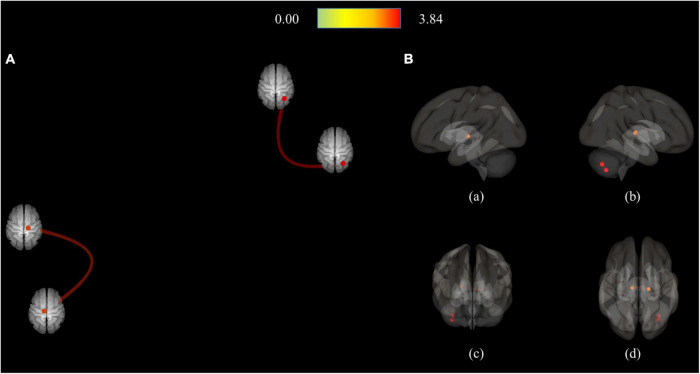
Connection pairs of HC < MDD functional connectivity within 71 ROIs for the ROI-to-ROI analyses. **(A)** ROI-to-ROI connectome ring maps. **(B)** ROI-to-ROI 3D rendering maps on the left (a), right (b), anterior (c), and superior view (d). The color bar indicates the statistical *T*-value. HC, Healthy controls; MDD, Major depressive disorder; ROI, Region of interest.

In contrast, in the MDD group, only four pairs of FC showed higher connectivity than the HC group, which included Crus II of the right cerebellum (R-Cerebellum_Crus2)–R-Cerebellum_7b, R-Cerebellum_7b–R-Cerebellum_Crus2, right ventral posterolateral thalamus (R-Thal_VPL)—left thalamus intralaminar (L-Thal_IL), and Thal_IL-R–Thal_VPL (Figure 4 and Table 4).

**TABLE 4:** Connected pairs showing significant differences in functional connectivity on HC < MDD contrast.

Connection pairs			Statistic T (df = 82)	p-FDR
		
Seed regions		Target regions		
R-Thal_VPL	–	L-Thal_IL	3.38	0.0386
L-Thal_IL	–	R-Thal_VPL	3.38	0.0386
R-Cerebellum_Crus2	–	R-Cerebellum_7b	3.84	0.0085
R-Cerebellum_7b	–	R-Cerebellum_Crus2	3.84	0.0085

df, degree of freedom; FDR-corr, False Discovery Rate corrected; HC, Healthy controls; L-Thal_IL, Left intralaminar thalamus; MDD, Major depressive disorder; R-Cerebellum_Crus2, Crus II of the right cerebellum; R-Cerebellum_7b, Lobule VIIB of the right cerebellum; R-Thal_VPL, Right ventral posterolateral thalamus.

## Discussion

According to the seed-to-voxel analysis results of this study, the MDD group exhibited significantly reduced RSFC in areas including the L-Habe, L-Cerebellum_6, Vermis_7, and R-Cerebellum_6, compared to the HC group. However, no significant difference between the groups of R-Habe or Sep_N was found in RSFC. In the ROI-to-ROI analysis using subcortex areas, including the habenula, brainstem, and cerebellum, as areas of interest, RSFC decreased among many subcortex areas in the MDD group.

To the best of our knowledge, this is the first study to identify a decreased association between the left habenula and cerebellum in depression. RSFC with other areas was analyzed using habenula as a seed in untreated patients with first-episode MDD patients previously, but the habenula and cerebellum areas did not show any significant RSFCs, and the area with the significant difference in RSFC between the patient and the control groups was the right dorsolateral prefrontal cortex area (53). In addition, the habenula area is a critical area for emotional processing, and the cerebellum is also widely involved in psychopathology in the psychiatric field (56).

It has also been reported that it is involved in the emotional process through structural and functional connectivity with various brain areas in depression (32). Previous brain functional imaging studies have also revealed that the activity of the limbic circuit is not regulated in patients with depression due to decreased activation of the prefrontal cortex, dorsolateral prefrontal cortex (DLPFC), and ventromedial prefrontal cortex (VMPFC); decreased blood flow; and decreased metabolism (57, 58). Consequently, it was assumed that the inability to process emotional stimuli appeared as clinical symptoms of depression, such as negative emotions, rumination, and decreased executive function (57, 58). However, a few studies have clearly revealed the connectivity between these substructures in MDD.

In particular, there are few studies on the connectivity between the habenula and other areas in depression. Previous studies reported the association between the habenula and striatal regions, midbrain, cortical regions, limbic structures, and insula in the normal population (59, 60). Wu found that habenular functional connectivity is positively correlated with ACC and PFC (53). Luan found increased connectivity between the right habenular nucleus with the anterior cingulate cortex, medial superior frontal gyrus, and medial orbitofrontal gyrus and decreased connectivity with the corpus callosum in the treatment-resistant depression group (61). They also found increased functional connectivity of the left habenula with the inferior temporal gyrus and decreased functional connectivity with the insula in treatment-resistant depression (61). Qiao found that patients with MDD displayed increased static FC from the habenula to the putamen but a decreased static FC to the precentral gyrus (62). In addition, their study found a decreased dynamic FC from the habenula to the angular gyrus (62).

The seed-to-voxel analysis showed no difference between the right habenula and other ROIs and RSFC groups. In animal studies, including fish, amphibians, reptiles, and vertebrates, habenula previously showed left-right asymmetry in habenula structures (63–66). In a human autopsy study, the habenular volume was larger on the left side in both genders (67). The left-right asymmetry in habenula volume in MDD was also shown in a structural study using seven Tesla MRIs performed by our previous research (68), and the number of right habenula-left mediodorsal thalamus tracts was higher in patients with MDD than in HC in a diffusion tensor imaging study (69). As a possible hypothesis for the asymmetry of functional connectivity in the habenula, structural (size) asymmetry could induce functional asymmetry, and structural asymmetry might facilitate more accurate and rapid control over binary behaviors such as escaping or freezing (70). Additionally, functional lateralization of the habenula may allow greater degrees of freedom regarding information processing when controlling social interactions and complex behavioral situations (71).

In the ROI-to-ROI analysis, the MDD group showed 31 pairs of decreased functional connectivities between ROIs compared to the HC group, and both Habe had decreased functional connectivity with the cerebellum. On the other hand, in the MDD group, only four pairs of increased functional connectivities were observed compared to the HC group. Since previous studies have never investigated RSFC using the only subcortical area as ROI in patients with depression, no previous study is comparable to this study. However, a previous whole-brain resting-state analysis showed decreased functional connectivity of affective and cognitive networks in medication-free patients with major depression (72). In a meta-analysis study on RSFC in MDD, seeds related to processing emotion or salience, including the cerebellum, mainly showed decreased RSFC and hyperconnectivity in the default network (73).

In MDD, RSFC increased only between the seed and the adjacent ROI, showing increased connectivity in the cerebellum area and only FC within the thalamus area (Figure 3 and Table 4). On the other hand, reduced RSFCs in MDD were also found between areas separated from each other. In addition to the cerebellum and thalamus, RSFCs significantly decreased in MDD were also found among the vermis, substantia nigra, red nucleus, and ventral tegmental area (VTA). Specifically, it was found that the remaining 30 RSFCs, excluding right Thal_MDm–right thalamus reuniens (Thal_Re) connectivity, correlated between inter-regional connections (Figure 2 and Table 3).

In the depression group, many cerebellum (particularly vermis area) areas showed reduced association with other ROIs. The FC between L-habenula and Vermis_7 was significantly increased in HC compared to MDD in the ROI-to-ROI analysis, as in the seed-to-voxel analysis results. Of the 31 pairs of RSFCs reduced in depression, 30 included the cerebellum area. L-Cerebellum_9 had the most FCs and targeted ROI regions, including the thalamus, Red_N, SN_pr, and R-habenula. Direct comparison is impossible because there are few results from previous studies in the same area. However, in studies on RSFC, subjects with MDD and those at high risk for MDD showed significantly decreased ReHO in the regional homogeneity (ReHO) of the whole brain study (31), and the cerebellum exhibited hypoconnectivity with the posterior parietal cortex in the meta-analysis (73). Using the cerebellar seed-based method, Guo et al. insisted that decreased cerebellar-DMN coupling was associated with treatment resistance of MDD using the cerebellar seed-based method (74). Lai and Wu found a decreased inter-hemispheric connectivity in the anterior sub-network of the default mode network and the cerebellar posterior lobe in MDD (75). Ma et al. suggested that the altered cerebellar–cerebral RSFC could be used as classification features to discriminate MDD patients from HCs.

Although new findings were reported in this study that may contribute to understanding the neural circuitry of depression, it is not without limitations. First, in the MDD group, there were key differences such as the severity of depressive symptoms, remission status, duration of depressive episodes, the factor of whether or not medications were taken, and the types of medications taken, all of which could have been reflected in the outcomes by affecting brain function (76, 77). Second, the number of cases was too small to generalize these results. Third, we adopted the head motion exclusion criteria, as in previous studies in estimating the artifact by the head movement (53); however, this should be further evaluated with the most appropriate threshold value (78). Fourth, the results from correlation analysis of bilateral habenula and septal nuclei cannot be obtained because they were segmented in a standard-space template image. To provide more reliable segmented ROIs, reliability analysis is necessary for further study. Lastly, as this study performed seed-to-voxel and ROI-to-ROI analyses using Pearson’s correlation, the connectivity intensity between the two areas could be determined; however, the causal influence between the two areas could not be analyzed. In future studies, the causal influence between regions should be investigated through effective connectivity analyses (79).

## Conclusion

In summary, this is the first study that determined the observation of lower RSFC found among the ROIs of the subcortical areas in patients with MDD. If the newly reported abnormality of the neural circuitry in MDD is continuously revealed in future studies, it might contribute to elucidating the etiology of MDD. For functional connectivity to function as a functional brain biomarker for MDD, a further replication study is required, and the establishment of large data sets with comparable study methods will be an essential part.

## Data availability statement

The raw data supporting the conclusions of this article will be made available by the authors, without undue reservation.

## Ethics statement

The studies involving human participants were reviewed and approved by the Institutional Review Board of the Gil Medical Center. The patients/participants provided their written informed consent to participate in this study.

## Author contributions

C-KK and S-GK contributed to the conception and design of the study. S-GK organized the database. J-YJ, NK, C-KK, and S-GK performed the statistical analysis. J-YJ, S-EC, and S-GK wrote the first draft of the manuscript. J-YJ, S-EC, C-KK, and S-GK wrote sections of the manuscript. All authors contributed to manuscript revision, read, and approved the submitted version.

## Footnotes

1
www.nitrc.org/projects/conn

2
http://www.mccauslandcenter.sc.edu/mricro/mricron/

## Supplementary material

Supplementary Figure 1 Segmented habenula and septal nucleus indicated with a red overlaid on the T1 template image. **(A)** Segmented bilateral habenula on axial, coronal, and sagittal views. **(B)** Segmented septal on axial, coronal, and sagittal views.
