# Supplementary figures and images for: Decreased resting-state functional connectivity of the habenula-cerebellar in a major depressive disorder
Source: Front Psychiatry. 2022 Sep 6;13:925823. doi: 10.3389/fpsyt.2022.925823 (PMC9485485; doi:10.3389/fpsyt.2022.925823)

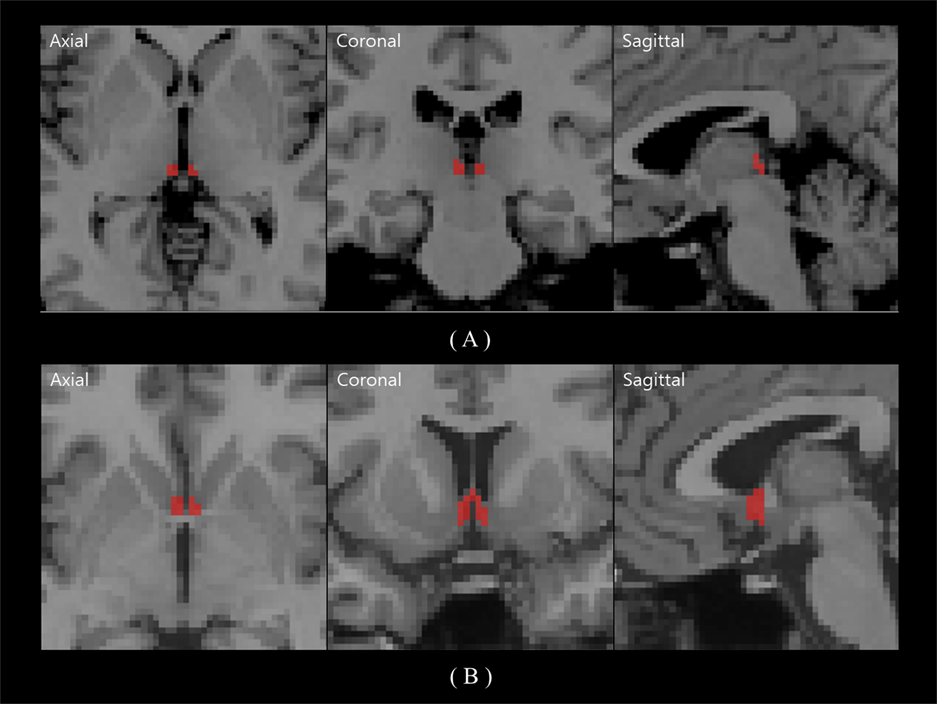

Supplement: Supplementary Figure 1 — Segmented habenula and septal nucleus indicated with a red overlaid on the T1 template image. (A) Segmented bilateral habenula on axial, coronal, and sagittal views. (B) Segmented septal on axial, coronal, and sagittal views. [file Image_1.TIF]
